# Expression pattern of protein kinase C δ during mouse embryogenesis

**DOI:** 10.1186/1471-213X-13-2

**Published:** 2013-01-10

**Authors:** Sergio Carracedo, Ursula Braun, Michael Leitges

**Affiliations:** 1 The Biotechnology Centre of Oslo, University of Oslo, Gaustadalleen 21, Oslo, N-0349, Norway

**Keywords:** Novel protein kinase C, PKC delta, Mouse embryogenesis, Lac Z, Ganglia

## Abstract

**Background:**

The members of the protein kinase C (PKC) family consist of serine/threonine kinases classified according to their regulatory domain. Those that belong to the novel PKC subfamily, such as PKCδ, are dependent on diacylglycerol but not Calcium when considering their catalytic activity. Although several studies have shown the importance of PKCδ in different cellular events in health and disease, the overall in vivo distribution of this PKC isoform during development is still lacking. Through Lac Z and antibody staining procedures, we show here the in vivo expression of PKCδ during mouse embryogenesis.

**Results:**

Ganglia were the domains with most prominent expression of PKCδ in most of the stages analysed, although PKCδ could also be detected in heart and somites at earlier stages, and cartilage primordium and skin among other sites in older embryos.

**Conclusions:**

The strong expression of PKCδ in ganglia during murine development shown in this study suggests a significant role of this isoform as well as redundancy with other PKCs within the nervous system, since PKCδ deficient mice develop normally.

## Background

In mammals, the PKC family consists of at least 10 serine/threonine kinases grouped into three subfamilies attending to their regulatory domain and requirements for their activation. Unlike conventional PKCs, novel PKCs (nPKC), such as PKCδ, are not dependent on calcium but on diacylglycerol to change from their self-inhibited state to their active conformation [1]. PKCδ activity can be regulated in different manners, including phosphorylation of its activation loop by kinases [2] and by autophosphorylation of different sites throughout its regulatory domain and hinge region [3]. In addition, it can become a lipid-independent enzyme with altered substrate specificity under certain conditions [3] and show altered cofactor requirements [4]. PKCδ interacts with different proteins, such as Shc [5] or p23 [6], and in its active state is able to phosphorylate different substrates, such as STATs [7] or ERK [8]. Functionally, PKCδ regulates different processes, such as cell cycle (by either slowing [9,10] or promoting [8,11] cell proliferation, depending on the context), apoptosis [12], cell migration [13] or transcription [14]. Also, although PKCδ deficiency does not prevent mouse viability [15], this isoform appears important in different contexts in health, as in the immune system [16] or insulin sensitivity [17], and disease, as for example in arteriosclerosis [15]. However, data regarding the general PKCδ expression pattern during mouse embryogenesis are still missing. This study shows the spatiotemporal expression of PKCδ during midgestation by immunostaining wild type mouse embryo sections and by using PKCδ deficient embryos expressing the LacZ reporter gene under the control of the endogenous PKCδ promoter. These results could be helpful when addressing functional redundancy or exclusiveness of this nPKC during murine development.

## Results and discussion

### PKCδ expression from E8.5 to E9.5

**Figure 1:**
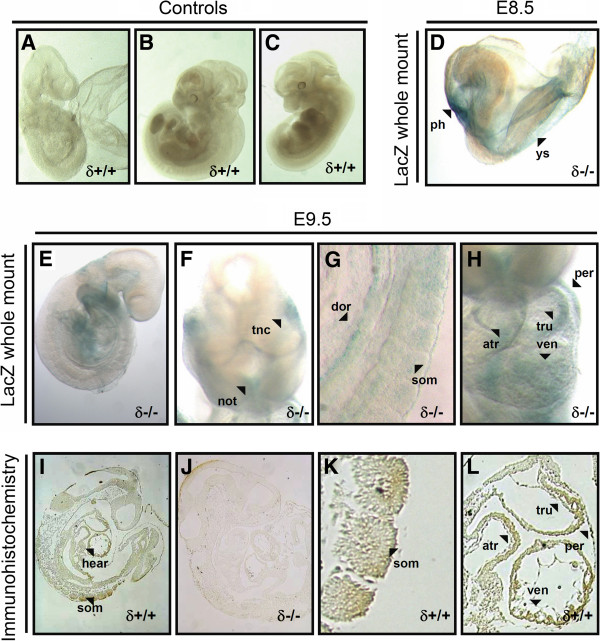
**PKCδ expression at E8.5 and E9.5. A-C**, lack of signal due to endogenous β-galactosidase was confirmed by applying the same LacZ staining protocols on wild type embryos (controls). **D**, at E8.5, X-Gal staining is detected in the yolk sac and primitive heart (ys and ph, respectively). **E-H**, at E9.5, trigeminal (V) neural crest tissue (tnc), rostral extremity of the notochord (not), dorsal aorta (dor), somites (som), pericardium (per) and developing heart all showed LacZ signal. In the latter, reporter activity was detected at the walls of the primitive ventricle (ven), atrium (atr) and truncus arteriosus (tru). **I-L**, Antibody staining of 4 μm E.9.5 embryo sections confirmed the expression of PKCδ in heart and somites. E9.5 PKCδ deficient embryo sections (**J**) were used as a negative control for the antibody.

PKCδ analysis was mainly performed through LacZ staining. Antibody staining with the corresponding negative controls was also performed at E9.5 and E13.5 together with the LacZ staining method as a control for the specifity of the LacZ signal. To confirm the absence of endogenous β-galactosidase activity in our stainings, wild type littermates underwent the same protocol in parallel (Figures 1A-C).

At E8.5, whole mount LacZ staining showed signal mainly at the primitive heart and yolk sac (Figure 1C).

Whole mount LacZ staining of PKCδ deficient embryos at E9.5 showed signal in rostral extremity of the notochord, trigeminal (V) neural crest tissue, dorsal aorta, pericardium, and developing heart. A closer look to the heart allowed for visualization of walls of the primitive ventricle, atrium and truncus arteriosus (Figures 1E-H). Immunostaining of wild type embryo sections at E9.5 confirmed expression of PKCδ in somites and the same areas of the heart and pericardium (Figures 1I-L). The specificity of the signal given by the antibody was confirmed by using PKCδ deficient embryo sections corresponding to the same developmental stage (Figure 1J).

### PKCδ expression from E10.5 to E12.5

**Figure 2:**
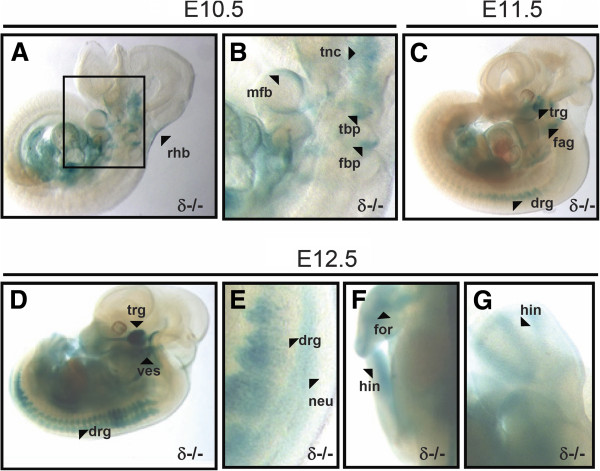
**PKCδ expression in whole mount embryos from E10.5 to E12.5. A** and **B**, at E10.5, roof of the hindbrain (rhb), third branchial pouch (tbp), fourth branchial pouch (fbp) and mandibular component of the first branchial arch show novel LacZ reporter activity. Signal at the trigeminal (V) neural crest tissue (tnc) became more prominent than at E9.5. Figure **B** is a close-up of the inset found in fig. **A**. **C**, At stage E11.5, staining of the trigeminal (V) ganglion (trg), facio-acoustic (VII-VIII) ganglion complex (fag), and dorsal root ganglia (drg) appeared all LacZ stained. **D-G**, 12.5 dpc embryos show increased signal in dorsal root ganglia (drg), strong LacZ activity in the trigeminal ganglion (trg), and novel activity at vestibulocochlear ganglion (ves), neural tube (neu) and precartilage primordia of bones at forelimbs (for) and hindlimbs (hin).

At E10.5, novel β-galactosidase activity was observed at the roof of the hind brain, third branchial pouch, fourth branchial pouch and mandibular component of the first branchial arch (Figures 2A and B). The signal observed at E9.5 in trigeminal (V) neural crest tissue became more prominent at E10.5 (Figures 2A and B). E11.5 was the earliest developmental stage at which ganglia started to show Lac Z reporter signal. Thus, dorsal root ganglia, facio-acoustic (VII-VIII) ganglion complex and trigeminal (V) ganglia all displayed β-galactosidase activity (Figure 2C). In 12.5 dpc embryos, dorsal root ganglia showed increased LacZ staining, and the trigeminal (V) ganglion became also prominently stained (Figures 2D and E). In addition, novel signal was detected at this stage in the vestibulocochlear ganglion (Figure 2D) and neural tube (Figure 2E). The broad and strong LacZ reporter activity detected in ganglia suggests that PKCδ may have a significant role within the nervous system in mice. However, there is no obvious defect within the nervous system during murine development and adulthood. We believe this is due to functional redundancy among members of the PKC family. For example, overlapping expression of PKC δ [18] and ε [19] within the nervous system can be already found as part of a recent study that uses an *in situ* hybridization approach to show the expression pattern of a high number of transcripts in the mouse embryo [20]. Thus, a redundancy where these two isoforms compensate each other could be a reason why no obvious phenotype is observed in the nervous system of PKC δ or ε single deficient mice during mouse embryogenesis.

At 12.5 dpc, embryos also showed novel reporter activity at the precartilage primordia of bone at forelimbs and hindlimbs, such as femur and radius (Figures 2F and G).

### PKCδ expression at embryonic stages E13.5 and E14.5

**Figure 3:**
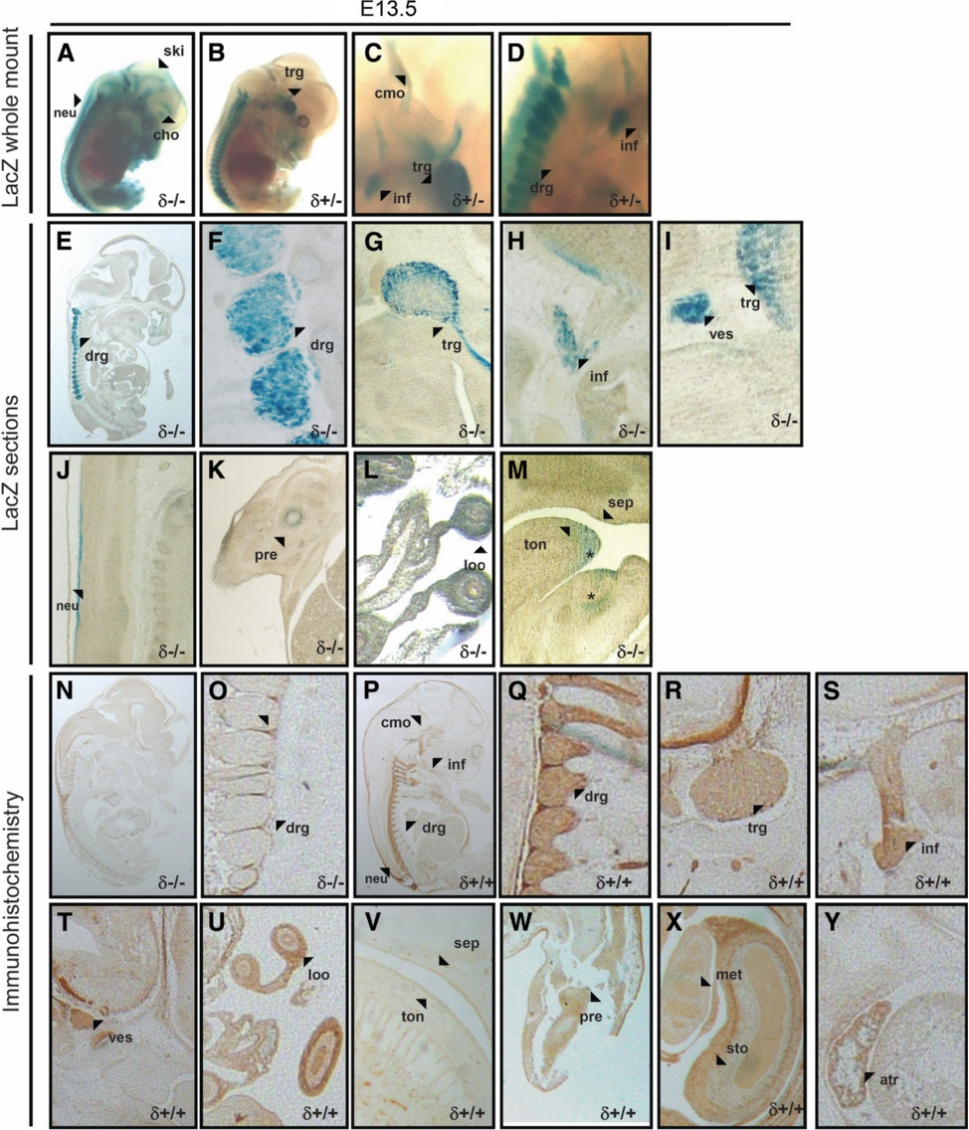
**PKCδ expression is highest within the nervous system at E13.5. A-D**, at embryonic stage E13.5, whole mount staining allowed for the detection of LacZ signal in skin (ski) in homozygous but not heterozygous embryos. Choroid plexus (cho), neural tube (neu), caudal region of the medulla oblongata (cmo), inferior ganglion of glossofaringeal (XI) nerve (inf), trigeminal (V) ganglion (trg), and dorsal root ganglia (drg) all showed novel β-galactosidase activity in whole embryos. **E-M**, 15 μm sagittal sections were obtained from LacZ stained embryos. Reporter activity observed in most cells of dorsal root ganglia (drg), trigeminal (V) ganglion (trg), inferior ganglion of glossofaringeal (XI) nerve (inf), vestibulocochlear ganglion (ves) and neural tube confirmed the signal observed in LacZ-stained whole embryos. Also, LacZ reporter activity was detected at cartilage primordium at limbs (pre), the loop of midgut within physiological umbilical hernia (loo), dorsal part of tongue (ton), and lower border of nasal septum (sep). Areas labelled as * could be detected only via LacZ staining and thus were not reported. **N** and **O**, at E13.5, PKCδ deficient mouse sections (δ−/−) were used as negative controls for immunohistochestristry (A and B). **P**-**Y**, at E13.5, antibodies to PKCδ confirmed LacZ staining at dorsal root ganglia (drg), trigeminal (V) ganglion (trg), inferior ganglion of glossofaringeal (XI) nerve (inf), vestibulocochlear ganglion (ves), loop of midgut within physiological umbilical hernia (loo), dorsal part of tongue (ton) and lower border of nasal septum (sep). Cross sections allowed for immunodetection of PKCδ in cartilage primordium at limbs (pre), metanephros (met) and wall and mucosal lining of the stomach (sto). In addition, antibody staining was detected in the atrium of the heart (atr).

**Figure 4:**
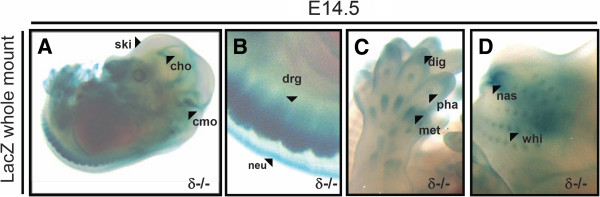
**PKCδ expression at E14.5. A-D**, in 14.5 dpc embryos, increased LacZ activity was detected in choroid plexus (cho), caudal part of medula oblongata (cmo), skin (ski), dorsal root ganglia (drg) and neural tube (neu). More defined signal than at previous stages was also found in precartilage primordia of digit (dig), precartilage primordial of phalangeal bone (pha) and metatarsal bone (met). LacZ reporter activity was also observed in whiskers (whi) and precartilage primordium of nasal septum (nas).

At E13.5 (Figure 3), dorsal root ganglia showed approximately the same strong LacZ signal observed in trigeminal (V) ganglia (Figures 3A-D). New domains with β-galactosidase activity at this stage of development were the caudal part of the medulla oblongata, inferior ganglion of glossofaringeal (XI) nerve, skin, and choroid plexus (Figures 3A-D). However, LacZ signal in the latter two domains was not detectable in PKCδ+/− embryos (Figure 2B). At this stage, LacZ-stained embryos were also embedded in paraffin blocks to generate sections that could let us better identify domains where β-galactosidase activity occurred. Given the low signal observed in the 4 μm-thick sections, 15 μm sections were used instead in order to obtain a more prominent LacZ staining signal. Unfortunately, sections of such thickness affected somewhat the quality of the corresponding photographs. However, we were still able to identify domains that could also be observed in whole mount embryos, such as dorsal root ganglia, trigeminal (V) ganglion, vestibulocochlear ganglion, neural tube or cartilage primordium at limbs (Figures 3E-K), as well as new areas that we could not see in whole embryos, such as loop of midgut within physiological umbilical hernia, dorsal part of tongue and lower border of nasal septum (Figures 3L-M). At this stage, there seemed to be problems with penetration of X-Gal in the embryo and therefore proper detection of signal in several domains, such as trigeminal ganglion (Figure 3G). Furthermore, sites such as stomach, which appeared stained at E12.5 (data not shown), was not detectable at E13.5, possibly due to the same problem. We also performed immunostaining of PKCδ in wild type and PKCδ deficient (negative control) mouse embryo sections at E13.5, which confirmed its expression at sites already identified in LacZ stained embryos: dorsal root ganglia, inferior ganglion of glossofaringeal (XI) nerve, vestibulocochlear ganglion, trigeminal (V) ganglion, loop of midgut within physiological umbilical hernia dorsal part of tongue, lower border of nasal septum, and cartilage primordium at limbs (Figures 3N-W). In addition, antibodies to PKCδ applied on cross sections also revealed expression in the stomach and metanephros (Figure 3X). Sagittal sections reported the atrium of the heart, which could not be seen in LacZ stained embryos or sections, possibly due to penetration issues of X-Gal (Figure 4Y), as earlier mentioned. There were some areas detected through Lac Z staining that could not be detected via immunostaining. In these areas, the Neo cassette that was used to generate PKCδ deficient mice might have influenced the expression of PKCδ [21], although PKCδ might instead be too lowly expressed to see immunosignal with the protocol we used.

Consistent with previous mRNA studies at E14.5 [20], our LacZ staining of embryos at this stage showed signal in brain, neural tube and ganglia (Figures 4A and B). However, we also found β-galactosidase activity at sites that we already described at earlier stages in this study, but whose patterns have not been reported before in such work at the mRNA, such as skin (Figure 4A, which also appeared LacZ stained in heterozygous embryos, unlike at E13.5) or cartilage primordia of bones (mainly at limbs, Figure 4C). The staining in bone was more prominent and more defined than when identified at E12.5. Here, it could be readily observed in precartilage primordia of digit, precartilage primordial of phalangeal bone, and metatarsal bone (Figure 4C). In addition, 14.5 dpc embryos displayed LacZ rerporter activity at whiskers and precartilage primordium of nasal septum (Figure 4D).

## Conclusions

Our expression pattern for PKCδ during mouse midgestation suggests that several domains, such as cartilage primordium or skin, express this novel PKC isoform. However, the nervous system is the main site of expression for PKCδ. More specifically, dorsal root ganglia and trigeminal (V) ganglia are the domains where PKCδ seems to be most prominently expressed. Thus, these data suggest that PKCδ may have an important role within the nervous system in mice, as already suggested by studies in other species [22,23]. The fact that there is no reported phenotype or functional deficiency in the nervous system suggest the existence of functional redundancy among members of the PKC family. Thus, the expression pattern of PKCδ may contribute to address such redundancy in function as well as to identify domains causing potential lethality in mice lacking several PKC isoforms.

## Methods

### Animals and embryo collection

Generation of mice (129/SvPasCrl) carrying the mutated allele for PKCδ has been previously described [15]. All animal work was approved by the Folkehelse Institute, Oslo (Norway) and performed according to its institutional guidelines and to the rules and regulations of the Federation of European Laboratory Animal Science Association´s (FELASA). Pregnancy stages were assigned upon observation of vaginal plug at approximately midday, which was considered as E0.5.

### LacZ staining

Steps corresponding to fixation (4% paraformaldedyde in PBS) and washing/permeabilization (Na_2_HPO_4_ 85 mM, NaH_2_PO_4_ 16mM, MgCl_2_ 2mM, 0.01% Na-desoxycholate, 0.02% NP-40) were performed for either 5 min (embryos up to 9.5 dpc) or 15 min (embryos from 10.5 dpc) at room temperature. Upon isolation, embryos were fixed, washed three times, and incubated with gentle shaking and protected from light overnight at 37°C in staining solution (for 10 ml, 9.7 ml of washing solution, 200 μl of K_3_[Fe(CN)_6_] 0.5 M, 200 μl of K_4_[Fe(CN)_6_] 0.5 M, and 175 μl of 50 mg/ml X-Gal (Sigma-Aldrich) in DMSO were used). Next day, embryos were washed three times at room temperature and postfixed in 4% formalin in washing solution overnight at +4ªC. PKCδ −/− embryos (unless otherwise stated) were then passed into increasing concentrations of glycerol (25%, 50% and 80%) and photographed by using a Zeiss stereoscope equipped with camera and Axiovision software. LacZ stained embryos to be sectioned were instead postfixed in bouin’s solution (Sigma) the next day after β-gal staining, washed 3 times in PBS, passed into increasing concentrations of ethanol (30%, 50%, 70% and 100%, 2 washes per concentration), placed into a mix 1:1 of Ethanol-xylene, washed 2 times in xylene, and finally embedded in paraffin.

### Immunostaining

Paraffin embedded sections of 9.5 and 13.5 dpc embryos were dewaxed using the following routine: 2 washes in xylol for 10 min, 2 washes in absolute ethanol for 5 min, 1 wash in 70% ethanol for 2 min, and at least 5 min in distilled water. Sections were then boiled for two minutes in citric acid pH 6.0 for antigen retrieval, washed three times in PBS, bleached for 20 min with a mix of 30% peroxide, 1M HCl, and methanol with the ratio 1:1:100, respectively, and incubated overnight in rabbit polyclonal anti mouse PKCδ (C-17, Santa Cruz Biotechnology) in a 1:200 dilution in PBS containing 5% fetal calf serum (FCS). Next day, sections were washed in PBS and incubated for two hours at room temperature in goat antirabbit IgGs conjugated to horse radish peroxidase (HRP, Jackson Immunoresearch) in a 1:200 dilution in PBS containing 5% FCS. Detection of PKC δ was then analyzed using the DBA method according to the manufacturer’s instructions (Biogenex).

## Abbreviations

Dpc: Days post coitum; nPKC: Novel Protein Kinase C.

## Competing interests

The authors declare no competing interests.

## Authors' contributions

SC acquired, analysed and interpreted the data, and ellaborated the manuscript. UB helped performing some experiments. ML and UB generated PKCδ deficient mice. ML participated in the design and interpretation of the experiments, and helped to write the manuscript. All authors read and approved the final version of the manuscript.
